# Dataset of user interactions across four large pilots on the use of augmented reality in learning experiences

**DOI:** 10.1038/s41597-023-02743-6

**Published:** 2023-11-24

**Authors:** Ana Domínguez, Guillermo Pacho, Lisa Bowers, Fridolin Wild, Sarah Alcock, Giuseppe Chiazzese, Mariella Farella, Marco Arrigo, David Ross, Rita Treacy, Darya Yegorina, Eleni Mangina, Stefano Masneri

**Affiliations:** 1 Vicomtech Foundation, Basque Research and Technology Alliance (BRTA), Digital Media, San Sebastián, 20009 Spain; 2 The Open University, Milton Keynes, MK7 6AA United Kingdom; 3 Consiglio Nazionale delle Ricerche, Palermo, 90146 Italy; 4 Words Worth Learning, Nenagh, E45 F827 Ireland; 5 Cleverbooks, Dublin, D15 DP2T Ireland; 6 UCD, Dublin, Dublin, 4 Ireland; 7 Computer Languages and Systems Department, University of the Basque Country UPV/EHU, San Sebastian, 20011 Spain

**Keywords:** Education, Education

## Abstract

Augmented Reality in education can support students in a wide range of cognitive tasks–fostering understanding, remembering, applying, analysing, evaluating, and creating learning-relevant information more easily. It can help keep up engagement, and it can render learning more fun. Within the framework of a multi-year investigation encompassing primary and secondary schools across Europe, the ARETE project developed several Augmented Reality applications, providing tools for user interaction and data collection in the education sector. The project developed innovative AR learning technology and methodology, validating these in four comprehensive pilot studies, in total involving more than 2,900 students and teachers. Each pilot made use of a different Augmented Reality application covering specific subjects (English literacy skills, Mathematics and Geography, Positive Behaviour, plus, additionally, an Augmented Reality authoring tool applied in a wide range of subjects). In this paper, we introduce the datasets collected during the pilots, describe how the data enabled the validation of the technology, and how the approach chosen could enhance existing augmented reality applications in data exploration and modelling.

## Background & Summary

By applying Augmented Reality (AR) technology in an education setting, new applications emerge which can positively affect the process of teaching and learning for students in real-world situations^1–4^. Like all technology-enhanced learning, AR in education creates data trails^5^ from which teachers and researchers can glean insight into actual learner behaviour. For this, linking AR interactions with all Learning Analytics is paramount^6^ for tracking and analysis, drawing from a variety of tools and approaches.

Most notably, up until recently, this typically would involve SCORM^7^, a set of technical standards for e-learning products, which enable tracking of learners’ experiences at a rather high level. While the SCORM-provided granularity was sufficient for decades, today, as demands rise for educational data mining, data scientists and pedagogues are using machine learning techniques to analyse the learning experience, requiring more fine-grain, more in-depth, and larger volumes of data.

The Experience Application Programming Interface (xAPI, https://github.com/adlnet/xAPI-Spec) is an open e-learning specification, which allows recording nearly every type of learning activity. For example, it can track which buttons the user clicks or how long the learner spent reading a page. In order to do that, it collects data on the learner activity in real-time, storing it in an easy-to-read semantic string composed of three main constituents: *actor*, *verb* and *object* (for example, ‘Sally’ ‘played’ ‘the instructional video’).

Previously, the xAPI has already been used as a data collection system in AR, such as in ‘AugmaniaTM’^8^ which used learning activity data to display it as an interactive visualisation. It was also used in the area of Positive Behaviour Intervention and Support (PBIS)^9^, to study user interactions with augmented reality objects in behavioural lessons. Additionally, it has also been used to design frameworks for Learning Analytics^10,11^.

All the aforementioned examples foster data generation to facilitate the analysis of student behaviour in their e-learning activities. There is, however, a lack of datasets that allow analysing the effectiveness of AR in learning experiences, shedding light on how to reduce the complexity of authoring tools for AR applications. To address these gaps, we developed and piloted a set of AR applications, putting effort into tracking users’ interactions via the xAPI to evaluate the effectiveness of AR interactive technologies when being used by both students and teachers.

The data provided in this work has been collected in four different pilot studies. The documentation produced across the lifetime of the project, along with the datasets created, should enable researchers to re-use the datasets and/or the methodology in future projects. The first three pilots address learning activities to be experienced by students, while the last one tackles the authoring process of creating educational AR experiences for teachers:

- **English Literacy:** Consists of a study investigating how AR technologies can help students improve their English literacy skills^12^. This pilot tests an application provided by WordsWorthLearning (https://wordsworthlearning.com/). Several English teachers or Special Needs Assistants of pupils in the 4th—6th-grade primary school, who typically underperform in standardised English literacy tests, included this application in their lessons. 53 students participated in this pilot.
- **Science, Technology, Engineering and Mathematics (STEM) learning:** This study investigates the impact of AR technologies on STEM skills learning and retention^13^. The pilot used two AR applications, created by CleverBooks (https://www.cleverbooks.eu/), for learning geography and geometry. In this case, 128 primary school teachers who are actively involved in teaching mathematics or geography topics in the 4th and 5th grades of primary education have been recruited, and they included the apps mentioned above in their lessons. This pilot involved 2,504 students, across schools in 11 European countries.
- **Behavioural lessons:** This study aims to work with AR to practise expected student behaviour. Positive Behaviour Intervention and Support (PBIS)^14^ is a framework that originated in the United States for creating positive school climates. It supports establishing the social climate and individualised behaviour guidelines needed for a safe and effective learning environment for all students. To study the impact of AR technologies on behavioural lessons, this pilot provides an application developed by Consiglio Nazionale delle Ricerche (https://www.pa.itd.cnr.it/index.html) that has been tested with 189 students aged 9–12 in Italian and Dutch primary schools working with PBIS.
- **Learning eXperience Design (LXD):** aims to accelerate the uptake of AR in education, by providing an authoring tool and learning management system to teachers. The objective of this pilot was to quantitatively and qualitatively evaluate in which ways and how well the authoring toolkit supports teachers in designing Augmented Reality learning experiences. 109 teachers from 23 countries across Europe participated in this pilot.

We believe the datasets generated from these pilots will benefit researchers and educational staff wishing to demonstrate the advantages of using AR in education, showcasing effective ways of interacting with the augmented content. Moreover, the data gathered through applications in these pilots is complemented by questionnaires and interviews carried out before and after the pilots. These complementary data sets (and more) can be found in ZENODO (https://zenodo.org/communities/augmented) and Argos (https://argos.openaire.eu/explore-plans/publicOverview/c49291ee-ee2c-4b92-823e-be146dcf2410) repositories.

## Methods

Before focusing exclusively on the main contribution of this work, i.e. the data collection using xAPI, it is important to note that the pilots were carried out in the framework of the European project ARETE (https://www.areteproject.eu/project/), where the role of ethics has been fundamental. A suitably experienced External Ethics Advisory Board (EEAB) was recruited for the ethics requirements purposes of the project, with emphasis on the synthesis of expertise. *Anonymity*, *Confidentiality* and *Privacy* were three terms of high importance, which adhered to within the pilots and communicated to all participants so as to assure trust, transparency, reliability and integrity. Arrangements were in place to ensure that the identity of each participant remained confidential and recommendations of the European Group on Ethics in science and new technologies (EGE, https://research-and-innovation.ec.europa.eu/strategy/support-policy-making/scientific-support-eu-policies/european-group-ethics_en) were followed. Additionally, national and international regulations related to research in the project were observed, in particular the European Data Protection Regulation (GDPR, https://gdpr-info.eu/), which entered into force in May 25th 2018.

In short, all the pilots involved the collection of data from teachers, students, parents, administrators, and other stakeholders which were managed ethically providing information sheets and a consent form to each participant.

The process used to select the stakeholders involved in the studies was different in each case. However. all the pilots presented common aspects. Although all the participants in the pilots had previous experience with the subject being taught, in general they had no previous experience with AR applications for education. Apart from that, a common requirement for all the pilots was that teachers and students had have access to AR compatible Android or iOS devices.

The recruitment process for English Literacy and STEM pilots was conducted via a 2-step approach. The 1st step consisted in identifying teacher coordinators and the 2nd step consisted in recruiting pilot teachers. In both cases, to engage the highest number of teachers, an intense dissemination campaign took place via email and at events and meetings, as well as through industry partners, associations and other stakeholders usually involved in education projects. In addition, the network of European Schools was contacted and finally, a snowball approach was employed as selected teachers were also invited to contact and disseminate within their school and among their colleagues.

For the English Literacy pilot the inclusion criteria for students was that they had to be typically underperforming in English language literacy tests. Pilot sample consisted of 53 students (18 female and 35 male) whose age was between 9 and 13 years. Table 7 shows the geographic distribution of teachers and students in this pilot. As an incentive, participant teachers were given two test batteries to keep in their schools and students were given a year license to use the app. Teachers were also given a summary report on the progress of their class at the end of the project.

In the STEM pilot study, geometry and geography teachers with a good knowledge of English and students in grade 4 or 5 were required. Pilot sample consisted of 2504 students (1277 female and 1227 male) with an age range between 9 and 12. In this case, 1327 students were involved in the geometry pilot while 1177 participated in geography pilot. Table 8 shows the geographic distribution of teachers and students in this pilot. In this case, students were given free books and printed maps and teachers were also given a summary report on the progress of their class at the end of the project.

Regarding the recruitment process of PBIS pilot, schools in the Netherlands were recruited through a call for participants launched on social media and addressed to PBIS schools, as well as through the distribution of paper flyers directly to schools or at PBIS-related events. The Italian students were recruited from the only school working with PBIS in Italy, located in Palermo. Inclusion criteria for schools was the implementation of PBIS for more than one school year with fidelity. The inclusion criteria for students was to be in fifth or sixth grade students (age range: 9–12 years old). Pilot sample consisted of 284 students of which 189 students were assigned to the PBIS-AR group condition. From these 189 students, a total of 76 students completed the pre and post-test (36 females and 40 males). Table 9 shows the geographic distribution of teachers in this pilot. As an incentive, teachers were offered the opportunity to use the behavioral lesson package for free after the end of the project and students were rewarded with pins, anti-stress balls or T-shirts with the AR 3D character (ARpro coach) printed and a positive behavioral message on them.

Finally, for the LXD pilot, teachers were mostly recruited through the Scientix network (https://www.scientix.eu/), the community for science education in Europe managed by European Schoolnet. Information on the pilot goal, scope and requirements was circulated among Scientix members and then interested teachers were selected according to the selection criteria established: good knowledge of english; be pre- or in-service teacher; availability during the pilot; be living in any Horizon 2020 eligible country. Pilot sample consisted of 109 teachers (77 female and 32 male) aged 46.8 on average. Table 10 shows the geographic distribution of teachers in this pilot. In this case, no incentives were given to participants although the pilot was promoted as a *Free training course on an AR Authoring Toolkit*.

Returning to the monitoring of users activity during pilots, the AR apps used during the studies automatically collected user data. These applications implemented a tracking system to gather information about the interaction of students and teachers within the augmented environment. The tracking system is based on the IEEE xAPI, a software specification for a normative interface for data collection, which offers a set of rules that enables monitoring the interactions of a learner with the learning content. More specifically, the datasets were created using publicly available xAPI library implementations of the following versions:

- Javascript (https://rusticisoftware.github.io/TinCanJS/)
- ADL Unity3D (https://github.com/adlnet/Unity-xAPI-Wrapper)
- i5 toolkit (https://github.com/rwth-acis/i5-Toolkit-for-Unity)

The overall system is composed of two entities. The client entity generates the xAPI statements, and the Learning Record Store (LRS) is responsible for receiving the statements and storing them in a database. In this case, the client is the AR application used by students and teachers while the LRS used is Learning Locker (https://learninglocker.atlassian.net/wiki/spaces/DOCS), an open-source data repository that stores the learning activity statements collected via xAPI. All the statements generated from student interaction with the applications are stored in the Learning Locker platform, where they then can be explored using a web interface, or downloaded in CSV format to conduct a more detailed data analysis.

**Fig. 1:**
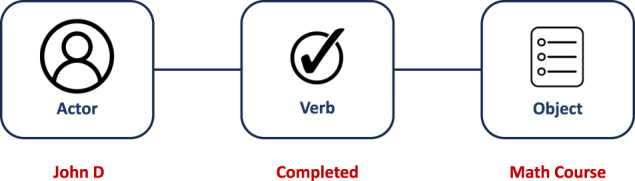
An example of xAPI statement.

Every xAPI statement is a JSON (JavaScript Object Notation) object. The basic structure of an xAPI statement takes the form of a triplet (Actor, Verb, Object). Actor refers to whom the statement is about, Verb is the action performed by such actor, while Object refers to the target, outcome, or result of the interaction. For better understanding, Fig. 1 depicts an example of an xAPI statement, referring to when a student completes a maths course. The actor, in this case, is the student ‘John’. The verb indicates that he is conducting an activity. The object defines what type of activity John is doing.

The statements can be customised, depending on the specific needs of the student or teacher, which gives the possibility to obtain additional information about the monitored activity. For instance, the timestamp specifies the exact moment in which the action was performed, the language field indicates the language in which students used the apps and a result field could also be added to include the score the students obtained in the corresponding activity. The main advantage of this tracking system is that it facilitates tracking the activity that a learner undertakes and, simultaneously, does this in a simple and consistent format.

All pilots described in this work used this data format to monitor student and teacher activity, but since the objectives of each pilot study were different, so is the data collected. The differences are based on two aspects: the vocabulary used and the nature of the data collected. Regarding vocabulary, different verbs and objects were selected depending on the activities and the 3D contents available in each app. As for the nature of data, we could discern the type of actions recorded, such as interactions with the app, interactions with other students in cases, where multi-user activities are provided, or logging data during the authoring process.

**Fig. 2:**
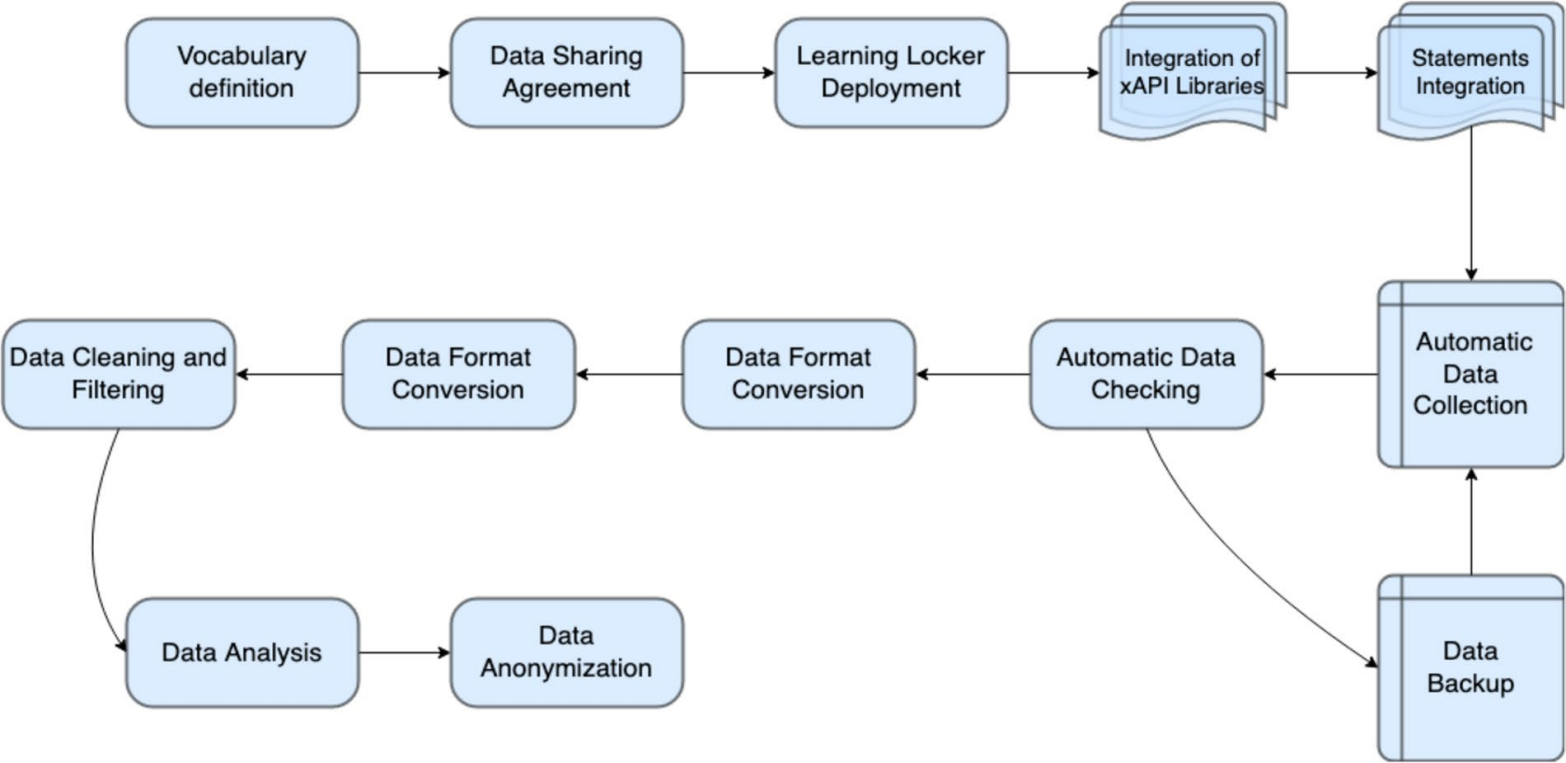
xAPI data workflow diagram.

Figure 2 shows the common steps undertaken for the overall workflow of the xAPI data capture. The first steps represent the set-up of the data collection system, from the creation of the vocabulary defining the set of statements, up to the integration and implementation into the frontend and backend. The following steps describe the automated data collection processes, data checking and backup. Finally, the last steps represent the processes before the publication of the datasets, namely data conversion, cleaning and filtering of the statements, exploratory and statistical data analysis, and data anonymization.

From this point, the data-collection process is introduced for each pilot, providing a description of each application and the list of statements defined for each case.

### English literacy pilot application

**Fig. 3:**
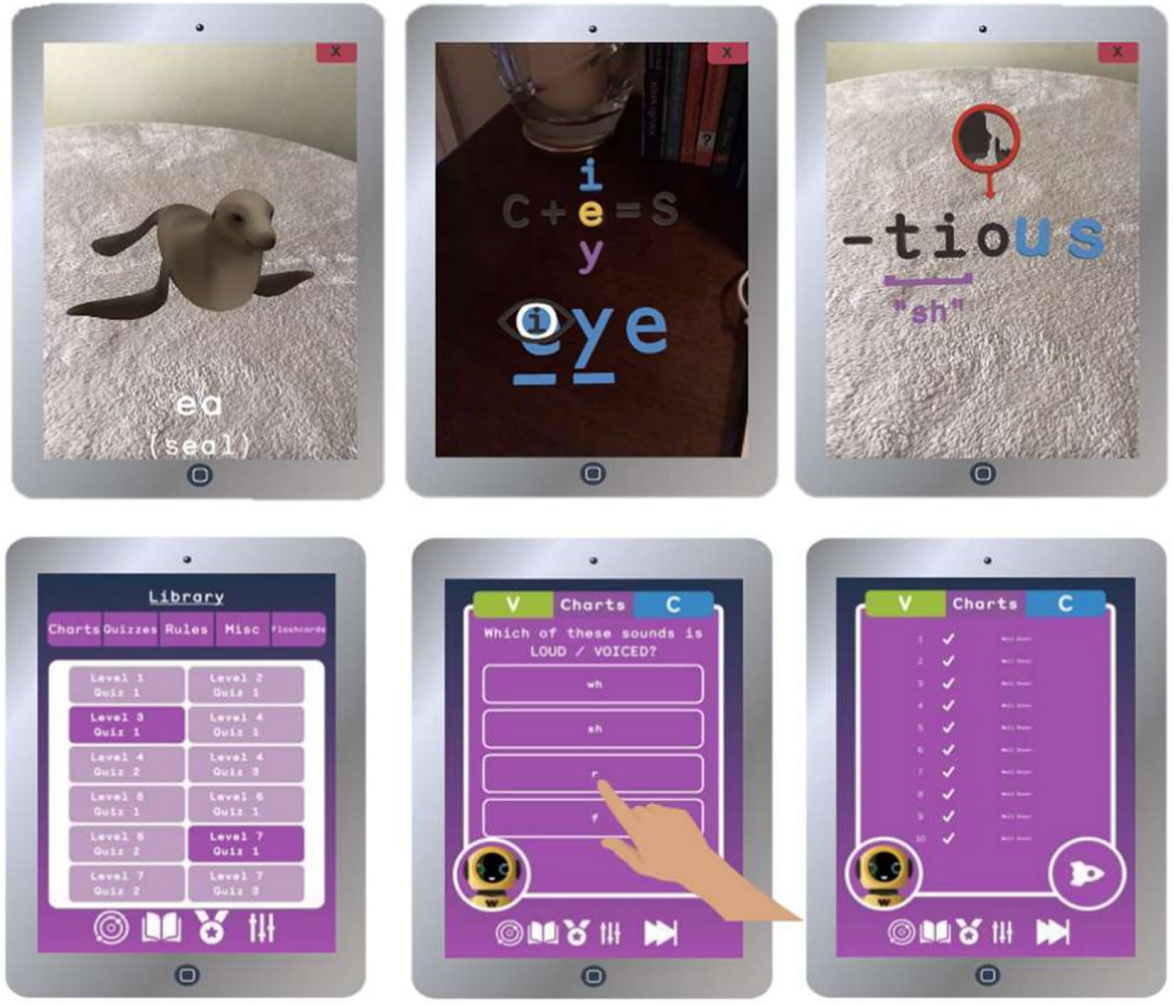
Screenshots of English Literacy pilot application.

In the case of the English literacy pilot study, the students used an AR application consisting of 7 different levels, related to reading and spelling activities. Each level contained different modules, and students had to complete each module before being able to start the following level. The students also had access to additional supporting material that enabled them to view charts on consonants and vowels and other types of AR contents. At the end of each level, students were evaluated on their comprehension with a ten-question multiple-choice quiz. To be able to proceed to the next level, students had to score ten out of ten on the quiz. Figure 3 shows some screenshots of the English Literacy application.

Table 1 shows the type of statements collected in the English Literacy pilot. This table can be seen as a summary of the verbs that are available in the corresponding dataset and their connection with the different objects within the application. These statements allowed the tracking of all the activity of each student within the app, taking into account their access to the app, the levels, modules, and exercises they carried out, the scores they obtained and the questions they succeeded or failed to answer.

**Table 1:** Type of statements gathered in English Literacy pilot.

Actor	Verb	Object	Notes
Student ID	Log in	Access app	
Student ID	Selected	Lesson	When students start a module within a specific level.
Student ID	Selected	Consonant chart	Students can check this chart to learn or review the pronunciation of different consonants.
Student ID	Selected	Vowel chart	Students can check this chart to learn or review the pronunciation of different vowels.
Student ID	Module status	Level	When students finish a module within a specific level.
Student ID	Enabled	App library	Students check the incorrect questions they got in a questionnaire.
Student ID	Pause app	Pause	When students pause the application
Student ID	Return app	Return	When students return to the application
Student ID	Attempted	Questionnaire	Students take questionnaires for knowledge check
Student ID	Attempted	SBLData	Students take spelling exercises based on SoapBoxLabs (paediatric Voice Recognition) API (https://docs.soapboxlabs.com/technical-docs/online-technical-documentation/verification-(online))
Student ID	Attempted	Reading	Students take reading exercises

### STEM pilot applications

In the case of the STEM learning pilot, we offered two applications (Geometry and Geography) with a similar structure. Unlike the English Literacy case, these applications did not have an established order for the students to follow. Students were provided with a book with different exercises that include continent maps in the case of geography and images of 2D and 3D shapes in the case of geometry. The images in the book were used as AR markers which enabled the applications to show the augmented contents.

#### Geography

**Fig. 4:**
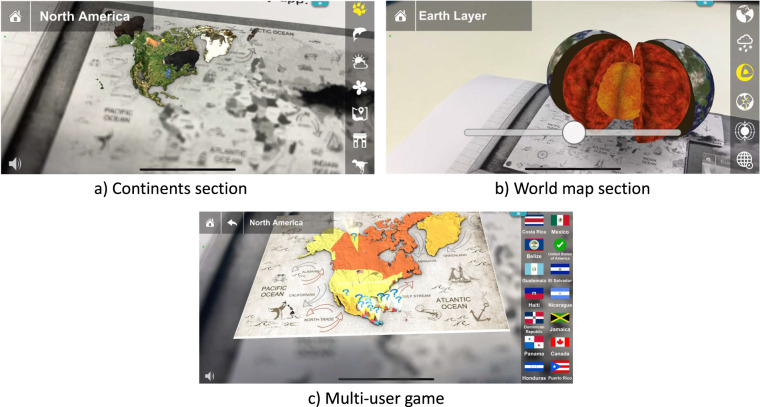
Screenshots of Geography application.

Within the geography application, there were three sections. The first one was related to continents (see Fig. 4a). Students were able to explore objects in the form of animals, plants, climate, heritage, and dinosaurs on a specific continent. When the student selected an object, a description was displayed. In addition, the student was invited to take a ten-question multiple-choice quiz individually. In the second section (see Fig. 4b) the students were presented with a world map designed to explore the earth, the weather, the layers and tectonic plates, magnetic fields or the timezone. The final section (see Fig. 4c) was intended to be a multi-user game in which students, in groups, had to match some objects to a place in the augmented map. For example, they were requested to place a flag on a specific country. In this case, the application, collected statements related to the interactions between different users, besides the interactions between the student and the app.

#### Geometry

**Fig. 5:**
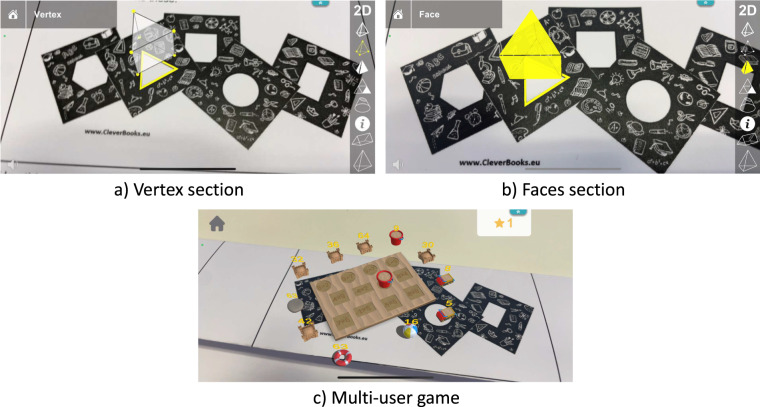
Screenshots of Geometry application.

In the geometry application, there were four sections. The first section (see Fig. 5a,b), was designed for students to interact with different 3D objects from solid geometry, facilitating understanding of these shapes and their constituents such as edges, vertexes, or faces. The second section contained a quiz of ten multiple-choice questions that allowed students to evaluate their knowledge of geometric shapes. This quiz could be repeated as many times as required. The questions varied and not all the students had the same set of questions to complete. The third section was an individual game in which students needed to correctly answer as many questions as possible, with the difficulty level increasing after every ten correct answers. Finally, the fourth section (see Fig. 5c) was a multi-user activity in which students practised and learned addition, subtraction, multiplication, and division operations combined with real-life objects in the form of figures.

Table 2 shows the types of statements collected in the STEM pilot, both in geometry and geography activities. This table can be seen as a summary of the verbs that are available in the corresponding datasets and their connection with the different objects within the application. These statements allowed the tracking of every interaction in the apps, from the access to each section to the interaction with the 3D objects available or the completion and scores in each quiz.

**Table 2:** Types of statements gathered in STEM pilot.

Actor	Verb	Object	Notes
Student ID	Selected	Geometry → A subsection in shapes section	User selected a specific subsection
		Geography → A subsection in continents section or elements in the augmented maps	
Student ID	Launched	Geometry → Section: AR scene, test, maths game or shapes game	User started a section
		Geography → Section: AR scene, test, game	
Student ID	Interacted	Geometry → objects showing different shapes	User interacted with 3D objects in multi-user games (moving objects)
		Geography → animals, flags, heritage, etc.	
Student ID	Completed	Geometry → test and shape games	User completed the test specified in the Object field
		Geography → test about animals, water animals, plants, weather, heritage, countries, dinosaurs	
Student ID	Answered	Answering a test question for geography and geometry	True or False

### Positive behaviour intervention and support pilot application

In the case of PBIS pilot, the application consisted of a mobile application with three different sections where students could complete activities related to what they learned in the behavioural lessons, both on their own or together with their classmates through multi-user activities.

**Fig. 6:**
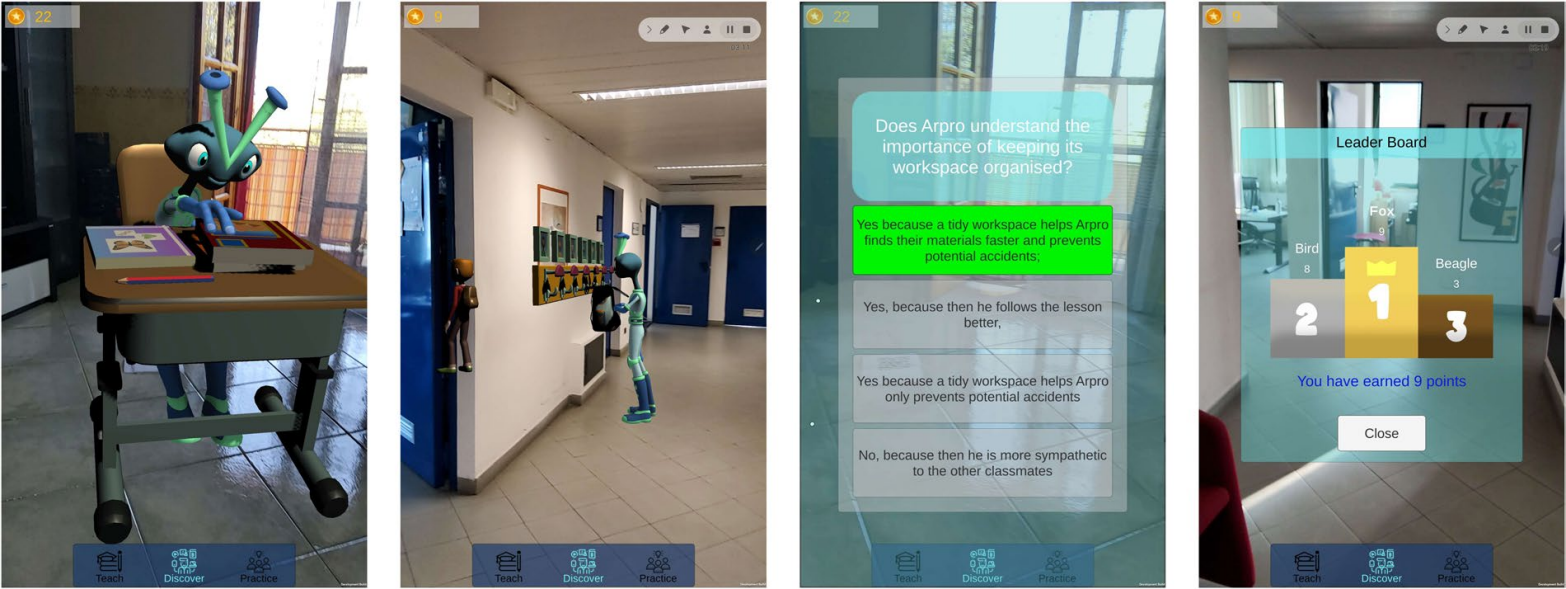
Discovery section of the PBIS app.

**Fig. 7:**
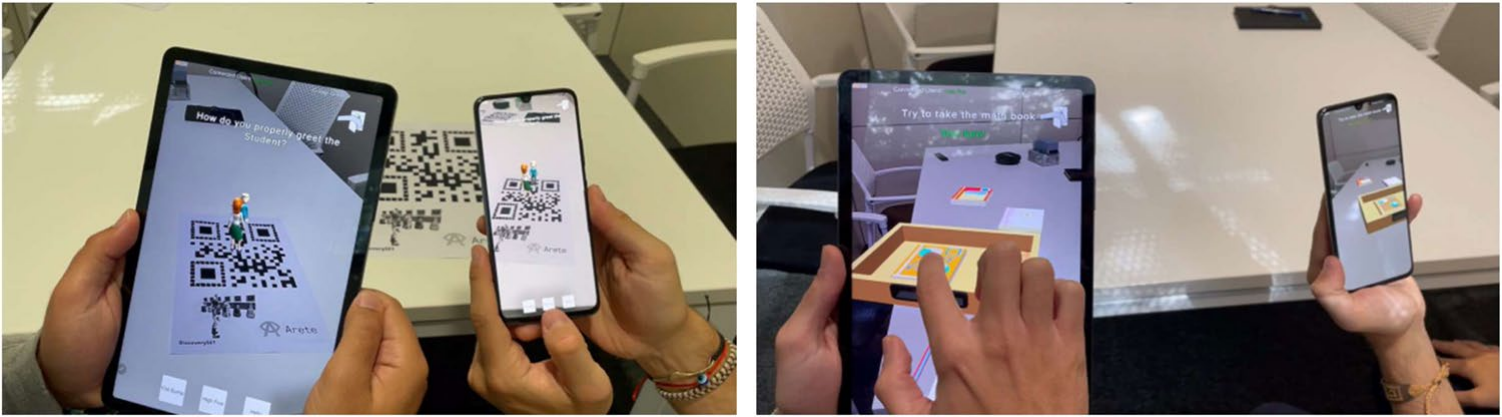
Practice section of the PBIS app.

Through the first section, called *Teach*, the student was able to visualise expected and unexpected examples of behaviour to reflect how to behave. The second section, called *Discovery*, allowed students to participate in a behavioural reflection game in AR, where they could test what they learnt about behaviour through a set of questions. A student can obtain a maximum of 3 points for a question answered correctly on the first attempt (3 correct; 1 partially correct; 0 incorrect). If the student answers partially correctly on the first attempt, a maximum of 2 points may be obtained (1 on the first attempt and a maximum of 1 on subsequent attempts answered correctly). Finally, if the user answers incorrectly on the first attempt, the student can obtain a maximum of 1 point by answering correctly on subsequent attempts. The result of questions provide an impact of the AR behavioural lesson on student behaviour learning but also the quality of student responses to each behavioural expectation. A series of markers located in different settings allowed showing the expected and unexpected behaviour of augmented characters on the screen, and then, the students were invited to reflect through quizzes. The AR contents were developed within 9 behavioural lessons, with an additional section, called *Practice*, that allowed the students to complete activities in pairs, each with their own device, interacting with the same augmented space. Table 3 lists all the behavioural lessons for which xAPI statements were collected, while Figs. 6, 7 show examples of the application.

**Table 3:** Behavioural lessons of the PBIS pilot.

Behavioural lesson	Multiuser?
I greet others	✗
I walk with a goal	✗
I keep my hands and feet to myself	✗
I keep my work space organised	✗
I clean up and store my belongings	✗
I work independently	✗
I stand up for others	✗
I help others with questions	✗
I let others be in peace	✗
Greet others	✓
Stand up for others	✓
Keep the workspace organised (untidy version)	✓
Keep the workspace organised (tidy version)	✓

Table 4 shows the types of statements collected in the PBIS pilot. This table can be seen as a summary of the verbs available in the corresponding datasets and their connection with the different objects within the application. This set of statements enables researchers to identify the activities carried out by students in all the different sections. The pedagogical value of introducing a set of xAPI for monitoring the student activity during the interaction with AR contents specifically developed for learning a specific behaviour is to understand learners’ interaction and preferences, and to assess the effectiveness of behavioural teaching intervention with the use of AR contents for PBIS. In this case, the set of xAPI statements allows monitoring the interaction with the coach Arpro (an alien character able to lead the student in learning the correct behaviours), collecting the answers given in the quizzes of the discovery section and the tasks carried out in the multi-user section where students in pairs and simulating a character role were asked to interact with objects and also answer some quizzes. Again, a series of primitives were defined to facilitate the tracking of these activities.

**Table 4:** Types of statements gathered in PBIS pilot.

Actor	Verb	Object	Result	Notes
Student ID	Started	Application		
Student ID	Read	Alien presentation		The user read the alien’s presentation
Student ID	Skipped	Alien presentation		The user skipped the alien’s presentation
Student ID	Selected	Teach/Discovery/Practice		The user selected the Teach, Discovery or Practice section
Student ID	Selected	Behavioural Lesson		The user selected the behavioural lesson from the UX wheel element
Student ID	Selected	Behavioural Resource		The user selected the AR resource of the behavioural lesson
Student ID	Selected	Multi-user Character		The user selected the character role
Student ID	Found	Discovery Marker		The user found the marker for AR discovery
Student ID	Found	Practice Marker		The user found the marker for AR multi-user practice task
Student ID	Completed	Discovery Resource		The user completed the AR Discovery resource
Student ID	Completed	Multiuser Task		The user completed the AR multi-user practice task
Student ID	Responded	Discovery Question	Response Raw score	The user selected the answer and was assigned a score (3 correct; 1 partially correct; 0 wrong)
Student ID	Responded	Practice Question	Response Raw score	The user selected the answer and was assigned a score (3 correct; 1 partially correct; 0 wrong)
Student ID	Joined	Practice Group		The user joined a multi-user group of Practice
Student ID	Accessed	Multi-user Task		The user accessed the multi-user task
Student ID	Left	Multi-user Task		The user left the multi-user group of practice
Student ID	Placed	Multi-user Resource		The user placed the object in the untidy or tidy AR drawer
Student ID	Consumed	Multi-user Task Attempts		The user consumed the attempts of a multi-user task

### Learning eXperience design pilot application

**Fig. 8:**
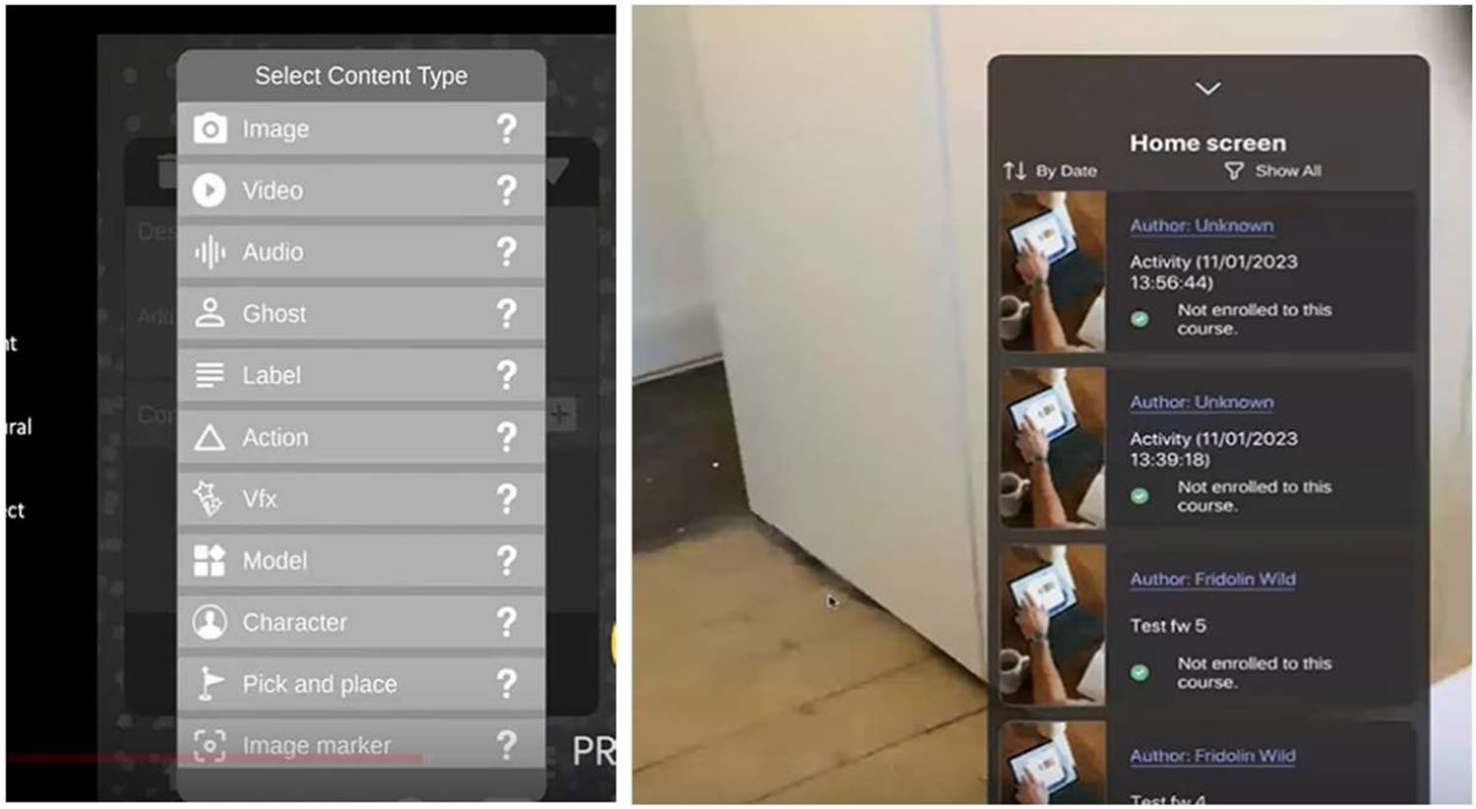
Initial content menu within the MirageXR application and overview of the Home screen.

In the case of Learning eXperience Design (LXD), the pilot focused on teachers’ use of an authoring tool (plus its interaction with a popular learning management system, Moodle). The tools were provided to the participating teachers to enable them to create AR learning experiences. The tools were integrated into the MirageXR platform^15^, an open-source application that allows creating AR learning experience without requiring any prior expertise in the use of AR. It provides users with a cross-platform authoring interface, through which participants of this study could design their own learning experiences. For the LXD pilot, participants could access Mirage XR version 1.9.2 on both iOS and Android devices, although the app is also available on both the HoloLens 1 & 2 (see Fig. 8). The target audience was pre-service or in-service teachers with a good level of English, and who had an AR-compatible iOS or Android tablet. The objective in the LXD pilot was to evaluate quantitatively and qualitatively the ways in which teachers used the authoring toolkit to support their design of XR learning experiences. The findings of the pilot lead to a reconceptualisation of the mobile user interface, resulting in the release of a major version 2.0.

For this study, participants were able to experience MirageXR in two modes 1) author modefacilitating teachers with designing and editing tools, 2) learner mode-aka play mode, which only permitted the learner to complete the LXD. Through toggling between modes, participants could design a learning experience as a non-expert, and then change to learning mode to understand the learning experience from a learner’s perspective. When in play mode, learners were limited to their engagement with predefined steps and interaction with objects (e.g., Pick & Place). However, Pick & Place holds great potential for the addition of assessment of the learners’ interactions, in that, learners can be examined on where they Pick and Place objects to a predefined location. If the objects are in the correct location, the learner will receive a rapid confirmation via audio feedback.

**Fig. 9:**
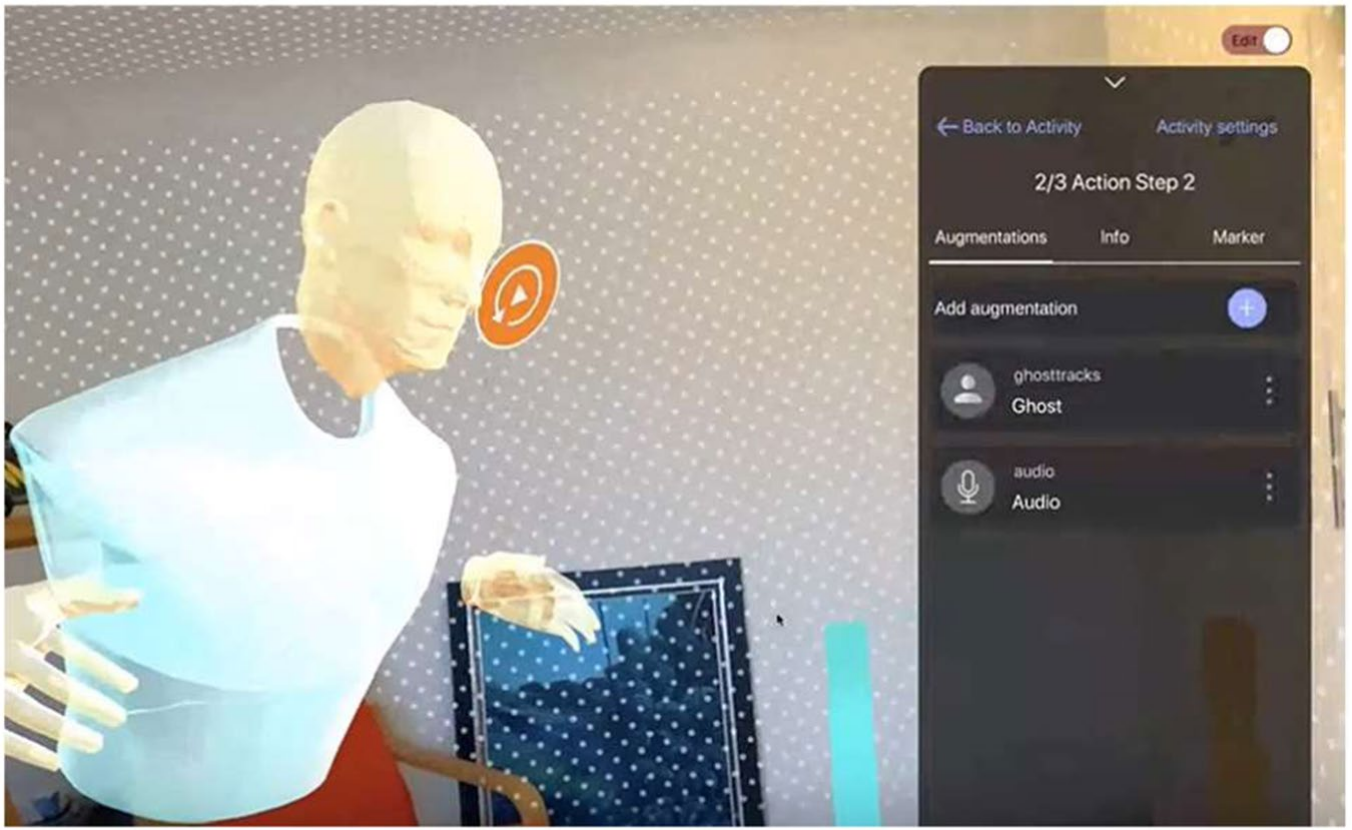
Ghost track SDK MirageXR in use.

For many learners, being ‘hands-on’ and actively engaging with a learning activity brings the learning to life. MirageXR offers a wealth of visual content in the form of 3D holographic, by using a holographic character, e.g., the Ghost track augmentation (depicted in Fig. 9). The addition of clear hands-on practice diminishes the need for text-based instructions or complex audio instructions that could be misconstrued, and instead, it offers a clear visual narrative, which the learner can mimic independently without the need for further assistance. Within the LXD pilot, MirageXR was used by teachers as an authoring tool to define, develop, and design learning experiences.

Teachers, without prior experience in using AR authoring tools, were shown step-by-step how to work through an AR content and LXD set-up and were then facilitated to design an LXD specifically for their learners. Teachers were to share their LXD with other teachers across 21 countries within Europe, via Moodle, thereby sharing ideas, and best practices for working with LXD.

Table 5 shows the list of statements collected in the LXD pilot. This table can be seen as a summary of the verbs available in the corresponding dataset and their connection with the different objects within the application. These Object ID and verb statements define the different characters and actions participants have undertaken within their (LXD). The verb statements indicate where the teachers added actions or focus points. The object ID, indicates which characters the teachers have selected to engage their students within the LXD content. MirageXR app offers a wide selection of animal, human and playful characters which are friendly hosts and assist the students to commence learning activities.

**Table 5:** Types of statements gathered in LXD pilot.

Actor	Verb	Object	Notes
Teacher ID	Viewed	Label, Image, model	The user viewed label, image and model
Teacher ID	Found	detect	The user found and detected an object
Teacher ID	Listened_to	audio, sound	The user listened to audio and sound
Teacher ID	watched	video	The user watched a video
Teacher ID	Focused_on	Act:highlight act:Point	The user focused on an action point which was highlighted to bring attention
Teacher ID	opened	Act:OpenBox	The user commenced with an action to open a box
Teacher ID	closed	Act:Closebox	The user commenced with an action to close a box
Teacher ID	packed	Act:pack	The user commenced with an action to packed a box
Teacher ID	unpacked	Act: unpack	The user commenced with an action to unpack a box
Teacher ID	picked	Act:pick	The user picked up an object
Teacher ID	placed	Act:placed	The user placed an object
Teacher ID	screwed	Act:screwed	The user action was screwed
Teacher ID	rotated	Act: Rotate	The user commenced with an action to rotate
Teacher ID	lowered	Act:lower	The user commenced with an action to lower
Teacher ID	located	Act:locate	The user commenced with an action to locate
Teacher ID	lubricated	Act:lubricate	The user commenced with an action to lubricate
Teacher ID	painted	Act:paint	The user commenced with an action to paint
Teacher ID	plugged	Act:plug	The user commenced with an action to plug
Teacher ID	unplugged	Act:unplug	The user commenced with an action to unplug
Teacher ID	unfastened	Act:unfasten	The user commenced with an action to unfasten
Teacher ID	measured	Act:measure	The user commenced with an action to measure
Teacher ID	notice	Vfx(vfx:*)	The user noticed an object
Teacher ID	launched	Activity - started	The user launched and started an activity
Teacher ID	initialized	Activity-loaded	The user initialised and loaded an activity
Teacher ID	completed	Activity-completed	The user started and activated a step
Teacher ID	started	Step activated,	The user started a step
Teacher ID	experience	Step deactivated	The user commenced with an experience and deactivated the step

The applications described were used by students and teachers of many European countries during different periods (between 2021 and 2023). Statements were gathered in four separate Learning Record Stores created through an instance of the Learning Locker deployed on AWS.

The datasets provided in this work are a simplified version of the information available at the Learning Locker. The following code listing shows an example of an xAPI statement received during the English Literacy pilot (with some fields removed for clarity). As can be seen, the statement is received as a JSON structure that, besides the *actor*, *verb* and *object* information, includes many additional metadata fields. The datasets include all the information stored in the Learning Locker instance but, during the data analysis conducted by researchers, the statements were filtered by removing all unnecessary or redundant information. This way, cleaner versions of the datasets which were simpler and faster to analyse were produced.


{ “stored”: “2022−09−30T13:34:35.959Z”, “active”: true, “client”: “60ffcf8d448b2d059a63e3c4”, “lrs_id”: “60ffcf8d448b2d059a63e3c3”, “statement”: {  “authority”: {  “objectType”: “Agent”,  “name”: “New Client”,  “mbox”: “mailto:hello@learninglocker.net” }, “stored”: “2022−09−30T13:34:35.959Z”, “actor”: {  “objectType”: “Agent”,  “name”: “1s1116”,  “mbox”: “mailto:student@app.com” }, “timestamp”: “2022−09−30T13:34:35.959Z”, “version”: “1.0.3”, “id”: “1e355533−6924−493b−8f25−246144adc23c”, “result”: {  “completion”: true }, “verb”: {


  “id”: “http://id.tincanapi.com/verb/selected/”,


  “display”: {   “en−US”: “Selected”  } }, “object”: {  “objectType”: “Activity”,


  “id”: “http://example.com/activities/student−lesson”,


  “definition”: {   “name”: {“en−US”: “Lesson”},   “description”: {“en−US”: “Level 1 Module8 started”}  } }},“voided”: false,


“verbs”: [“http://id.tincanapi.com/verb/selected/”],


“person”: {  “_id”: “6103e17eb196ba05dd9fdc52”,  “display”: “1−T−01−01”}, “timestamp”: “2022−09−30T13:34:35.959Z”, “organisation”: “60faab70448b2d059a63e375”,}


The cleaning process consisted of three steps. First, the relevant fields for learning analytics were filtered. In order to do that, the statements gathered at each pilot were analysed to avoid losing relevant data and a list of common fields was elaborated to have four datasets with the same structure.

Once the list was defined, Learning Locker was used to filter the data and export it to CSV format. Then, a Python script was used to process the files by removing unnecessary fields and simplifying the data, as in some cases the xAPI standard stores some fields as complex nested dictionaries. Finally, the data were reviewed to make sure that they comply with data protection regulations and to keep users’ identities confidential. This is in line with the ethics code used from the beginning to the end of each pilot study which underpins the development, dissemination, and research data analysis tasks. Throughout the pilots, the data gathered in the course of the research have been collected in such a way that the results cannot be traced back to individual participants.

## Data Records

Table 6 provides the references and the links to each dataset publicly available at Zenodo and some details about the data such as the file name, the number of rows in each dataset and the data collection dates. As can be seen, the English Literacy and PBIS pilots recruited a relatively small number of students in comparison with the STEM pilot, since the English Literacy pilot required primary school students who typically underperform in the English Language literacy test, and PBIS pilot required students enrolled in schools working with the PBIS framework. On the other hand, the STEM pilot requirements were only based on the age of the students and, since one of its objectives was to investigate the differences in students’ performances across different countries, it focused on recruiting students from as many European schools as possible. That is why the STEM dataset includes many more entries than the others.

**Table 6:** Links and general characteristics of the datasets.

Pilot	Repository	File Name	Number of rows	Data Collection Start	Data Collection End
English literacy	ref. ^16^	EnglishLiteracy-Anonymized.csv	33,628	11/11/2021	24/06/2022
STEM Geometry	ref. ^17^	STEMGeometry.csv	142,567	15/10/2021	14/07/2022
STEM Geography	ref. ^17^	STEMGeography.csv	136,416	15/10/2021	05/07/2022
PBIS	ref. ^18^	xAPI_PBIS.csv	12,390	19/10/2022	17/04/2023
Learning Experience Design	ref. ^19^	LXD.csv	5,142	13/06/2022	19/09/2022

**Table 7:** Geographic distribution of participants in English Literacy pilot.

Country	Number of participating teachers	Number of participating students
Ireland	6	40
Luxembourg	1	4
Italy	2	9
Total	9	53

**Table 8:** Geographic distribution of participants in STEM pilot.

Country	Number of participating teachers	Number of participating students
Spain	6	93
Italy	6	103
Greece	30	548
Serbia	16	272
Poland	8	130
Sweden	2	14
Portugal	10	201
Romania	8	202
Croatia	12	252
Turkey	28	642
Moldova	2	47
Total	128	2504

**Table 9:** Geographic distribution of PBIS-AR group participants in PBIS pilot.

Country	Number of participating teachers	Number of participating students
Italy	12	54
Netherlands	6	22
Total	18	76

**Table 10:** Geographic distribution of participants in LXD pilot.

Country	Number of participating teachers
Albania	1
Azerbaijan	1
Belgium	1
Bulgaria	1
Croatia	3
Cyprus	2
Czech Republic	1
Greece	24
Hungary	1
India	5
Israel	2
Italy	12
Kenya	1
Moldova	2
Netherlands	1
Palestine	1
Portugal	7
Republic of North Macedonia	3
Romania	14
Serbia	3
Slovakia	2
Spain	8
Trukey	13
Total	109

As mentioned in the previous section, all the datasets had a common structure which can be described as follows:

- **Timestamp**: timestamp in ISO 8601 format that represents the moment in which the statement was received at the database. The English Literacy pilot took place between November 2021 and June 2022. The STEM pilot between October 2021 and July 2022. The PBIS pilot took place from October 2022 to April 2023. Last, but not least, the LXD pilot took place between June and September 2022.
- **Lrs_id**: Learning Record Store in which the statement was received. All the statements in each dataset will present the same Lrs_id.
- **Actor name**: ID of the user interacting with the application
- **Verb id**: unresolvable internationalized resource identifier (IRI) of the action performed by the user. Note: The IDs look like uniform resource locators (URLs), but are not resolvable, i.e. they do not point to a corresponding web page, nor they represent a call to an external API.
- **Verb display**: human-readable description of the action performed by the user.
- **Object id**: ID of the element with which the actor interacts.
- **Object name**: name of the element with which the actor interacts.
- **Object description**: human-readable description of the characteristics of the element with which the user interacts.
- **Result**: shows the result obtained by the actor performing the corresponding activity, for example, the score in an exercise, or the successful completion of a section.
- **Language**: the language selected by the students to do the activities. It is only available in the STEM pilot since this pilot was carried out in schools in 11 different countries. Even though in the LXD pilot there were participants from 23 different countries, the pilot was always in English. The English Literacy pilot was conducted in English. While the PBIS pilot study was carried out in Italy and the Netherlands, there is no language recording field in the statements. However, it is possible to know whether a data item belongs to the Dutch or the Italian sample by taking into account the actor field. All strings that include the substring ‘klas[n]’ belong to the Dutch case, while ‘classe[n]’ is the substring for the Italian case.

## Technical Validation

The data provided in this work include usage data of very different apps, targeting very different concepts, but all of them are related to educational experiences. The first three pilots focus on AR learning experiences for students, tackling very different subjects such as English literacy, STEM, and PBIS, while the last one is focused on the data collected from an AR-based authoring tool that allows teachers to create their own learning experiences. The data collection has been performed automatically, as it relies on the inclusion of the xAPI libraries into the applications tested over the course of the four pilots. The data validation process has been performed in two separate phases: first over the duration of the pilots and afterwards to verify and standardise the format of the xAPI statements. To verify that the database was able to successfully collect all the statements, each day we performed a download of the statements sent to the LearningLocker instance associated with each pilot and made sure that no duplicate statements were generated and that the number of statements matched the sum of the statements sent by each client who used the AR application. Additionally, a Python script was used to check that the range of numerical data types fell within the expected range, the uniqueness of the statement ID, and the consistency of the ID and display values of the person field, to ensure that all the statements generated from a user map to a unique ID, even if the user accessed the app from different devices.

Once a pilot was over, we also performed a detailed analysis of the data that verified that all the expected types of statements were sent, that is, that each actor was able to send data and that all the domain-specific verbs defined in the xAPI vocabulary (https://www.w3.org/community/xapivocabulary/) were present in the dataset. The analysis also proved the time consistency of random subsets of the statements by verifying that the logical succession of correlated events was maintained (for example, we checked in the PBIS pilot that for the same Actor ID the Selected, Found and Complete verb appeared in that order) and that, for statements representing user actions to be performed sequentially, no other statements appeared in between them.

Additionally, we also verified that for each statement, the timestamp (representing when a statement was sent) and stored (representing when the statement was added to the database) values did not have a significant time difference, as it could potentially represent a server overload and the inability of the database to process all the incoming statements. For each pilot, the time delay between those values never exceeded five seconds, so ultimately no statement was discarded. Finally, with the aim of standardising the format across the different datasets, all dates were converted to ISO 8601 format, and all the textual fields (e.g., description inside of object) used American English as the only language option.

The data collected in each pilot are different both qualitatively (as each pilot defined its statements and focused on the collection of different information) and quantitatively since each pilot study had different requirements for student recruitment.

In spite of the mentioned limitations, these datasets may foster analysis in many dimensions. For example, based on the recorded timestamps, it could be studied how much time students spent in each type of activity, which could be an indicator of the difficulty of each activity but could also be related to the student’s interest towards the contents. It could also be possible to know in which order students did the activities or identify the moment of the day in which they were more efficient, e.g. in the morning or in the afternoon. Additionally, the number of times students exited the applications would deserve special attention, as it could be due to both a lack of interest and technical problems.

From the methodology point of view, these data could help to define what is required to track granularity and identify the behaviour of students while using an AR application and to specify the complementary information, such as questionnaires or interviews, that could be useful to evaluate the effectiveness of AR in terms of academic performance.

Well-structured datasets with a high number of entries could lead to the possibility of applying clustering techniques, to identify different behavioural trends or patterns, creating prediction systems taking into account the obtained scores up to a moment, or providing recommendations for extra activities to obtain better results. This high number could be achieved, for example, by consolidating the data collected from students in different classrooms or schools, or different students over the years.

The datasets provided are therefore considered as contributing to clarifying many unknowns and highlighting the benefits that augmented reality could have in the field of education.

## Usage Notes

The datasets are publicly available at ARETE Zenodo repositories for each pilot:

- English Literacy: https://zenodo.org/record/7876947/files/EnglishLiteracy-Anonymized.csv?download = 1
- STEM Geometry: https://zenodo.org/record/7877072/files/STEMGeometry.csv?download = 1
- STEM Geography:https://zenodo.org/record/7877072/files/STEMGeography.csv?download = 1
- PBIS: https://zenodo.org/record/7876959/files/xAPI_PBIS.csv?download = 1
- LXD: https://zenodo.org/record/8009365/files/LXD.csv?download = 1

Apart from the timestamp-based analysis mentioned above, many more conclusions could be drawn from the datasets provided. For example, a relevant aspect could be the language in which students used the apps since this could establish AR adoption rates in different countries. In countries where technology adoption rates in education are high, the use of AR apps for educational purposes may be more widely accepted and integrated into educational systems. In contrast, in countries where technology adoption rates are lower, there may be challenges with implementing the proposed methodology. Moreover, the availability of AR multi-user activities within the applications could lead to a comparison between the scores obtained when working alone and those obtained in a collaborative way. We also believe that the data provided in the datasets can help other researchers designing new pilot studies involving the use of technology in educational environments, by proposing an efficient, consistent and automated way to perform data collection of user interactions.

In order to carry out these analyses, the authors provide a python package (see Code availability section) with a set of functions to import and process xAPI statements and run some tests. This package is just considered as a helpful tool to face the analysis, but the data analysis could be based on Jupyter notebooks or other statistical software like R or SAS, using standard libraries or tools like Tableau for data visualization.

## Data Availability

The authors provide an open-source Python package (https://github.com/Stocastico/xapi_analysis) that simplifies the data analysis of datasets like the ones described in this manuscript. The library requires Python ≥3.9, Pandas and Seaborn, and it has been created using the nbdev v2 environment. The code for the applications used in English Literacy and STEM pilots will not be released as open source since the authors are planning to further develop and exploit them. Instead, two videos (English Literacy video, STEM video) are shared so that the reader can see how the applications were and how the students could interact with them. The code used for PBIS has been released under the EUPL license and the repository (https://gitlab.com/aretewp5/PBISAR-App) is available on Gitlab. And finally, the LXD pilot has been released with an MIT license and the repository (https://github.com/WEKIT-ECS/MIRAGE-XR/) is available on GitHub.
